# Supplementary material for: Small Heat Shock Protein 22 Improves Cognition and Learning in the Tauopathic Brain
Source: Int J Mol Sci. 2022 Jan 13;23(2):851. doi: 10.3390/ijms23020851 (PMC8775832; doi:10.3390/ijms23020851)
Supplement: Supplementary file 1 [file ijms-23-00851-s001.zip › ijms-1530719-supplementary.pdf]

Supplementary Information

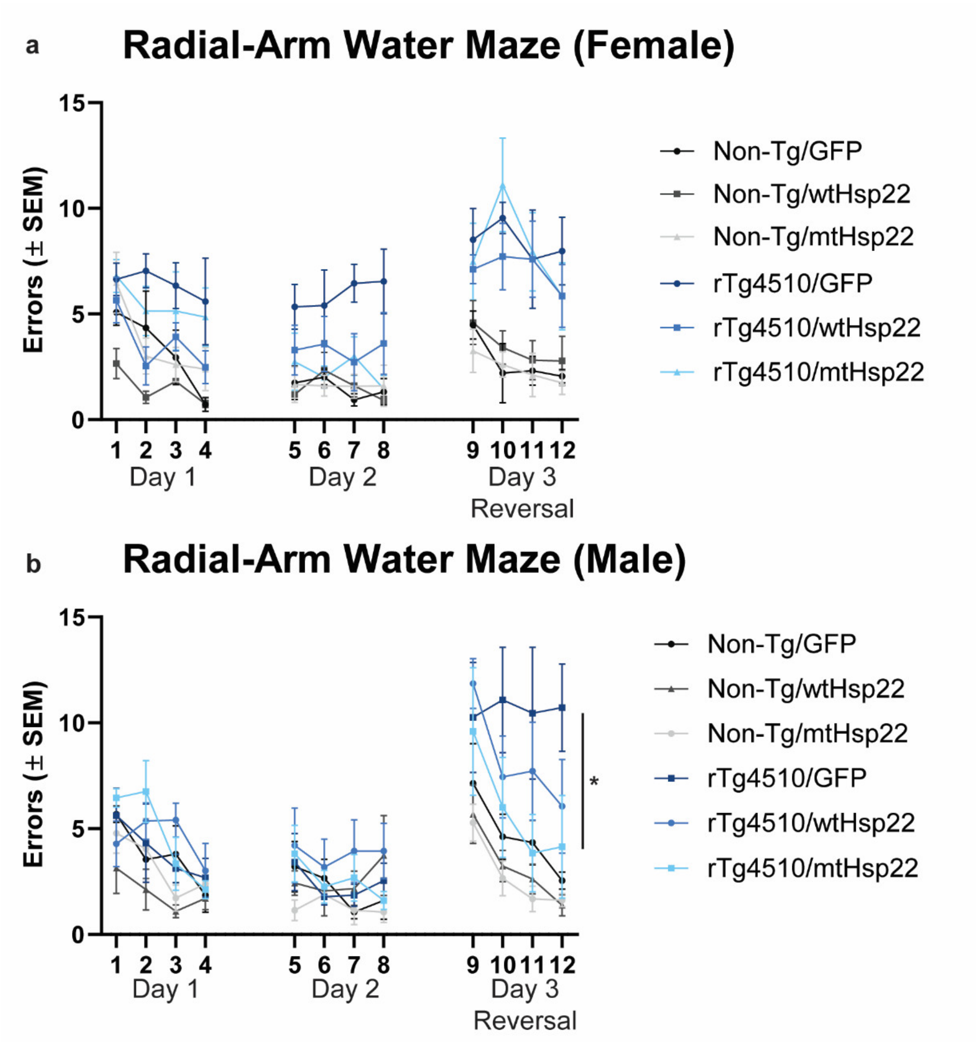

Supplementary Figure S1. mtHsp22 spatial learning and memory protection is greater in male rTg4510 mice. (**a**) Analysis of 2-day Radial-Arm Water Maze (RAWM) task with reversal (mean ± SEM) in female mice injected with GFP (*n* = 5/Non-Tg; *n* = 5 rTg4510), wtHsp22 (*n* = 5/Non-Tg; *n* = 5 rTg4510), or mtHsp22 (*n* = 5/Non-Tg; *n* = 5 rTg4510). (**b**) Analysis of 2-day Radial-Arm Water Maze (RAWM) task with reversal (mean ± SEM, *$p<0.013$) in male mice injected with GFP (*n* = 5/Non-Tg; *n* = 5 rTg4510), wtHsp22 (*n* = 6/Non-Tg; *n* = 5 rTg4510), or mtHsp22 (*n* = 5/Non-Tg; *n* = 5 rTg4510).

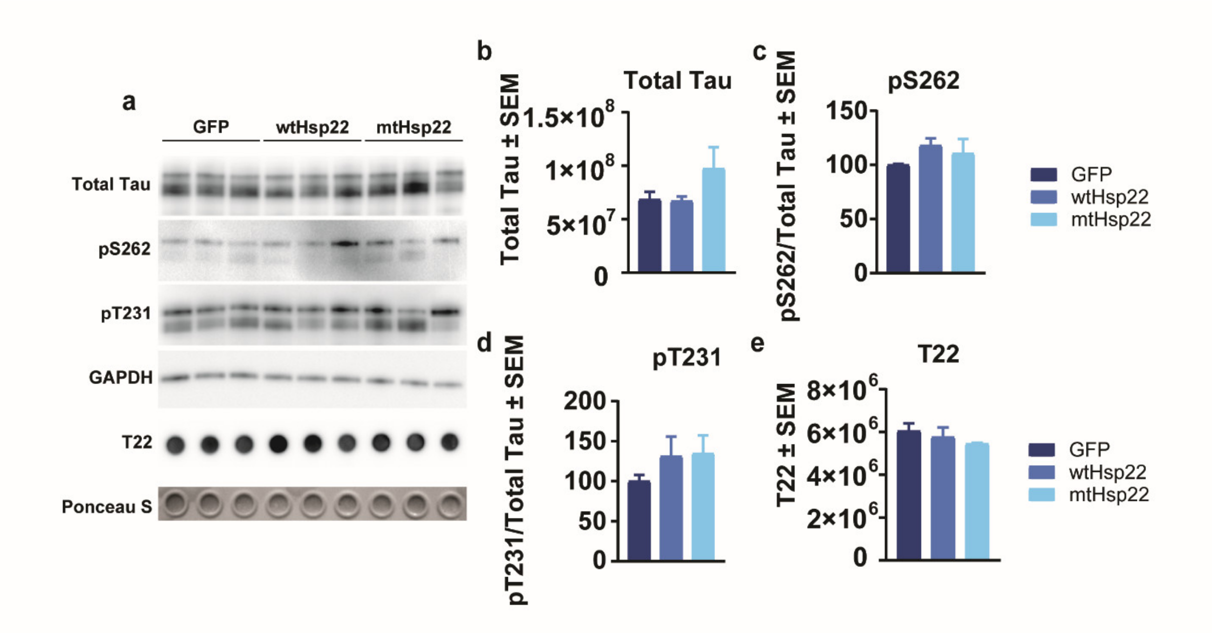

Supplementary Figure S2. Immunoblotting analysis demonstrates Hsp22 overexpression does not affect tau levels in rTg4510 mice. Immunoblotting and quantification of hippocampal lysates from rTg4510 mice overexpressing GFP (n = 3), wtHsp22 (n = 3), or mtHsp22 (n = 3); (**a**) Immunoblot of total tau, pS262, pT231 and T22. Quantification of (**b**) total tau, (**c**) pS262, (**d**) pT231 and (**e**) T22 (mean ± SEM).

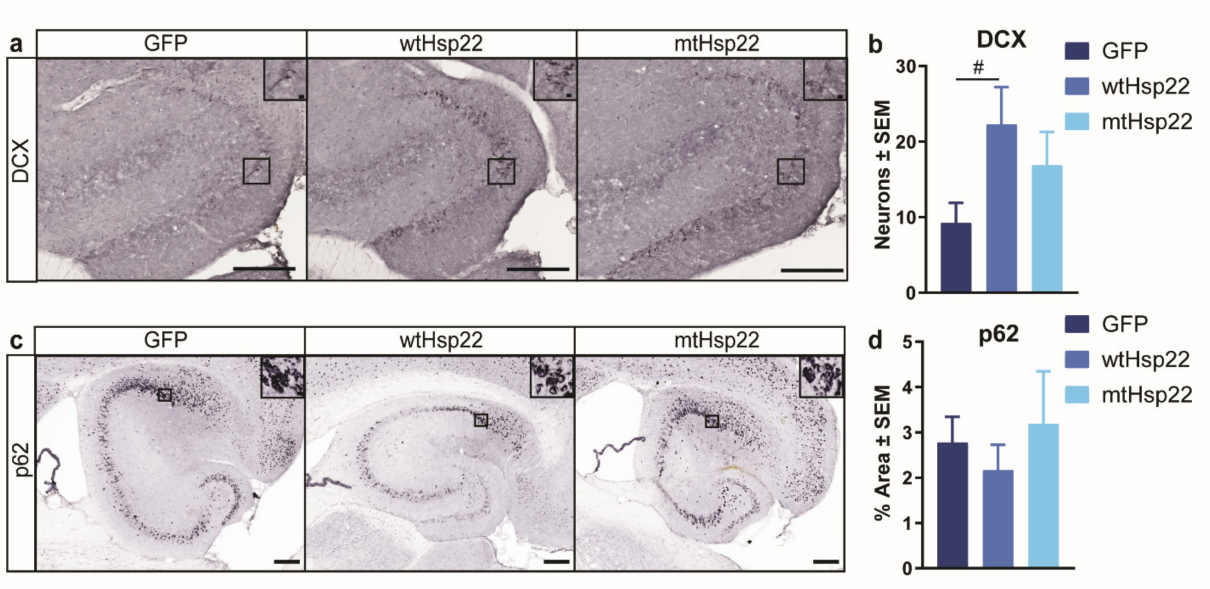

Supplementary Figure S3. Hsp22 overexpression does not affect hippocampal neurogenesis or markers of autophagy in rTg4510 mice. Immunohistochemical staining and quantification of the hippocampus from rTg4510 mice injected with GFP (n = 6), wtHsp22 (n = 6), or mtHsp22 (n = 6) for (**a**,**b**) doublecortin (DCX) (#p=0.749) and (**c**,**d**) p62 (mean ± SEM; scale bar represents 200 μm; inset represents 10 μm).

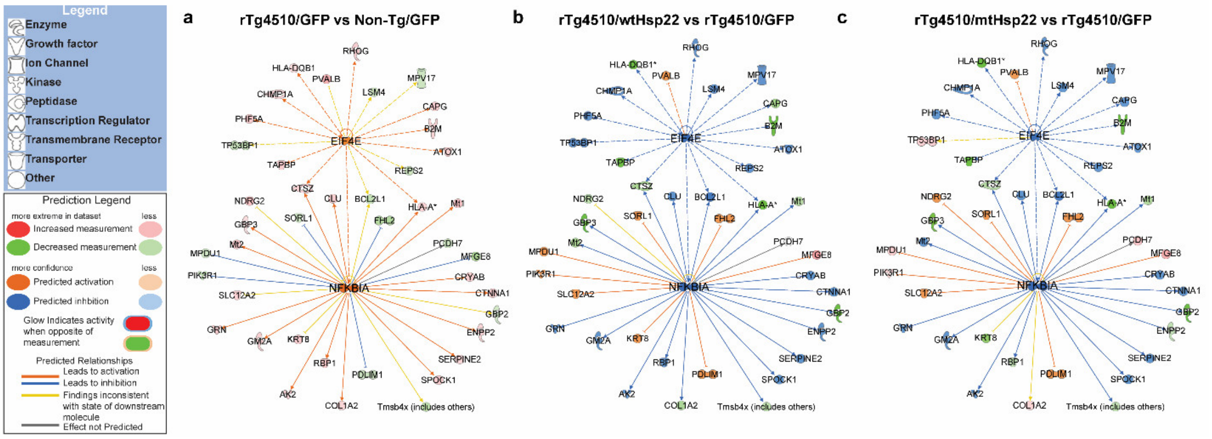

Supplementary Figure S4. Hsp22 overexpression shifts upstream regulators EIF4E and NFKBIA in rTg4510 mice towards non-transgenic levels. IPA network analysis featuring annotated upstream regulators EIF4E and NFKBIA, were fitted for comparisons; (**a**) *rTg4510/GFP vs. Non-transgenic (Non-Tg)/GFP* (**b**) *rTg4510/wtHsp22 vs. rTg4510/GFP*, and (**c**) *rTg4510/mtHsp22 vs. rTg4510/GFP*. Red and green nodes represent predicted activated and inhibited regulators, respectively. Orange and blue lines depict regulation from upstream molecule to downstream function.

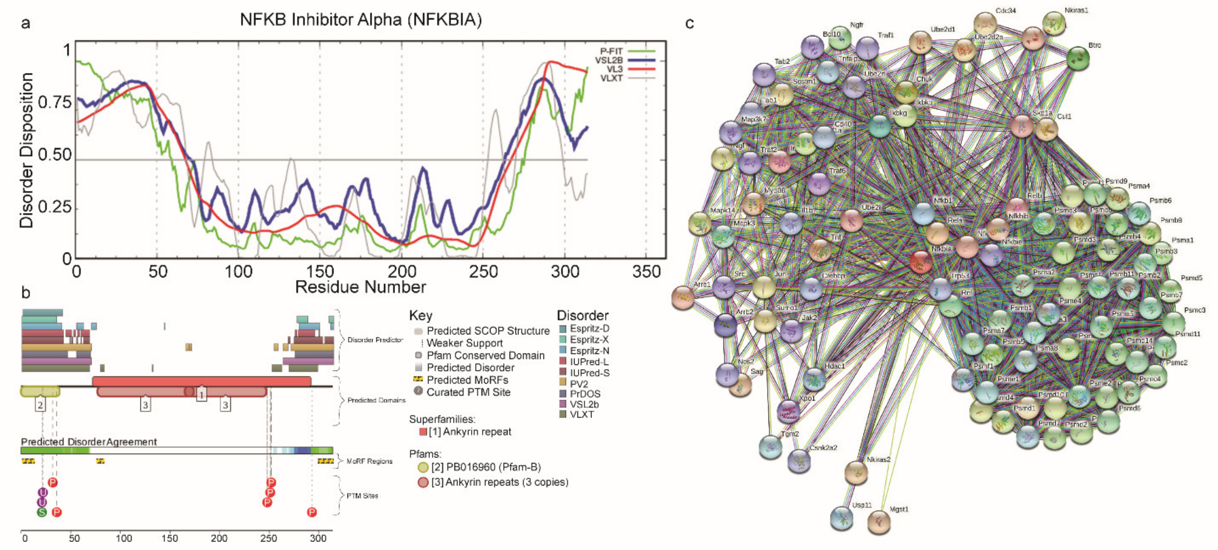

Supplementary Figure S5. Bioinformatics analysis of functional intrinsic disorder in mouse NFKBIA protein (UniProt ID: Q9Z1E3). (**a**) Intrinsic disorder profile generated by the DiSpi web crawler that aggregates the results from six commonly used disorder predictors: PONDR® VLXT [1], PONDR® VL3 [2], PONDR® VLS2B [3], and PONDR® FIT [4-6]. Mean disorder scores calculated by averaging the outputs of individual predictors together with the distribution of standard deviations are shown as well. The outputs of the evaluation of the per-residue disorder propensity by these tools are represented as real numbers between 1 (ideal prediction of disorder) and 0 (ideal prediction of order). A threshold of 0.5 was used to identify disordered residues and regions in query proteins. (**b**) Functional disorder profile generated for mouse NFKBIA generated by the D^{2}P^2 platform (http://d2p2.pro/) [7]. D^{2}P^2 is a database of predicted disorder for a large library of proteins from completely sequenced genomes [7] that uses outputs of IUPred [5], PONDR® VLXT [1], PrDOS [8], PONDR® VSL2B [2, 9], PV2 [7], and ESpritz [10]. The database is further supplemented by data concerning location of predicted disorder-based protein binding sites, known as molecular recognition features, MoRFs and various post-translational modifications. (**c**) STRING-generated PPI network of mouse NFKBIA. In this network, the nodes correspond to proteins, while the edges show predicted or known functional associations. Seven types of evidence are used to build the corresponding network, and are indicated by differently colored lines: green represents neighborhood evidence; red – the presence of fusion evidence; purple – experimental evidence; blue – co-occurrence evidence; light blue – database evidence; yellow – text mining evidence; and black – co-expression evidence [11].

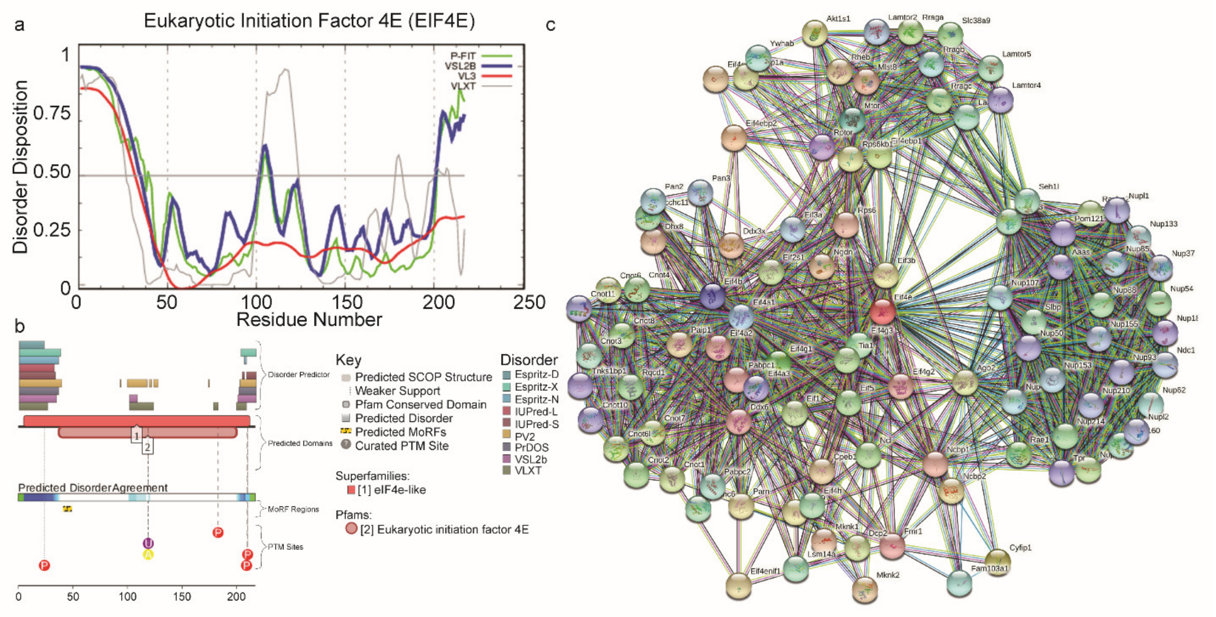

Supplementary Figure S6. Bioinformatics analysis of functional intrinsic disorder in mouse EIF4E protein (UniProt ID: P63073). (**a**) Intrinsic disorder profile of EIF4E generated by DiSpi. (**b**) D²P² generated functional disorder profile of human EIF4E indicating a high disorder level of this protein, the presence of one MoRF and multiple PTMs. (**c**) STRING-generated PPI network of mouse EIF4E. All the keys are the same as defined in Supplementary Figure S5.

Supplementary Information References

1. Romero, P.; Obradovic, Z.; Li, X.; Garner, E. C.; Brown, C. J.; Dunker, A. K., Sequence complexity of disordered protein. *Proteins* **2001,** 42, (1), 38-48.
2. Peng, K.; Radivojac, P.; Vucetic, S.; Dunker, A. K.; Obradovic, Z., Length-dependent prediction of protein intrinsic disorder. *BMC bioinformatics* **2006,** 7, 208.
3. Peng, K.; Vucetic, S.; Radivojac, P.; Brown, C. J.; Dunker, A. K.; Obradovic, Z., Optimizing long intrinsic disorder predictors with protein evolutionary information. *Journal of bioinformatics and computational biology* **2005,** 3, (1), 35-60.
4. Xue, B.; Dunbrack, R. L.; Williams, R. W.; Dunker, A. K.; Uversky, V. N., PONDR-FIT: a meta-predictor of intrinsically disordered amino acids. *Biochim. Biophys. Acta* **2010,** 1804, (4), 996-1010.
5. Dosztányi, Z.; Csizmok, V.; Tompa, P.; Simon, I., IUPred: web server for the prediction of intrinsically unstructured regions of proteins based on estimated energy content. *Bioinformatics* **2005,** 21, (16), 3433-3434.
6. Dosztanyi, Z.; Csizmok, V.; Tompa, P.; Simon, I., The pairwise energy content estimated from amino acid composition discriminates between folded and intrinsically unstructured proteins. *Journal of molecular biology* **2005,** 347, (4), 827-39.
7. Oates, M. E.; Romero, P.; Ishida, T.; Ghalwash, M.; Mizianty, M. J.; Xue, B.; Dosztanyi, Z.; Uversky, V. N.; Obradovic, Z.; Kurgan, L.; Dunker, A. K.; Gough, J., D(2)P(2): database of disordered protein predictions. *Nucleic Acids Res* **2013,** 41, (D1), D508-D516.
8. Ishida, T.; Kinoshita, K., PrDOS: prediction of disordered protein regions from amino acid sequence. *Nucleic Acids Res* **2007,** 35, (suppl_2), W460-W464.
9. Obradovic, Z.; Peng, K.; Vucetic, S.; Radivojac, P.; Dunker, A. K., Exploiting heterogeneous sequence properties improves prediction of protein disorder. *Proteins: Structure, Function, and Bioinformatics* **2005,** 61, (S7), 176-182.
10. Walsh, I.; Martin, A. J.; Di Domenico, T.; Tosatto, S. C., ESpritz: accurate and fast prediction of protein disorder. *Bioinformatics* **2012,** 28, (4), 503-9.
11. Szklarczyk, D.; Franceschini, A.; Kuhn, M.; Simonovic, M.; Roth, A.; Minguez, P.; Doerks, T.; Stark, M.; Muller, J.; Bork, P.; Jensen, L. J.; von Mering, C., The STRING database in 2011: functional interaction networks of proteins, globally integrated and scored. *Nucleic Acids Res* **2011,** 39, (suppl_1), D561-D568.
